# Comparative proteomic analysis of human serum before and after liver transplantation using quantitative proteomics

**DOI:** 10.18632/oncotarget.26761

**Published:** 2019-04-02

**Authors:** Ruohan Zhang, Minglin Ou, Yue Zhang, Qiang Yan, Huaizhou Chen, Liusheng Lai, Ying Li, Feilong Xu, Donge Tang, Xuyong Sun, Jianhui Dong, Yong Dai, Weiguo Sui

**Affiliations:** ^1^ Guangxi Key Laboratory of Metabolic Diseases Research, Nephrology Department of Guilin 924st Hospital, Guilin, Guangxi 541002, P.R. China; ^2^ Clinical Medical Research Center, The Second Clinical Medical College of Jinan University, Shenzhen People's Hospital, Shenzhen, Guangdong 518020, P.R. China; ^3^ Guangxi Key Laboratory for Transplantation Medicine Guangxi, Institute of Transplant Medicine, Department of Organ Transplantation in Guangzhou Military Region, 923st Hospital, Nannning 530021, P.R. China

**Keywords:** liver transplantation, proteomics, iTRAQ, protein markers, pathway

## Abstract

Liver cancer is the second leading cause of cancer mortality worldwide. Safer and more effective diagnostic methods for liver cancer are desirable, and biomarkers represent a potentially alternative method for diagnosis. The present study was designed to identify liver cancer biomarkers. We quantified the changes in serum protein levels between liver transplantation and healthy (control) females using isobaric tags for relative and absolute quantitation (iTRAQ) as well as proteomic analysis. A total of 1399 proteins were identified; of these, three proteins showed significantly different concentrations between the before transplantation group and the control group. These proteins may thus be relevant to liver cancer and constitute potential liver cancer biomarkers.

## INTRODUCTION

Liver cancer is the second leading cause of cancer mortality worldwide, causing more than 700,000 deaths annually [1]. Liver transplantation (LT) is the best curative treatment modality, achieving up to 91% 1-year survival post-transplantation [2]. However, finding the right donor liver is rare, and the patient must live as long as possible to wait for the liver from an appropriate donor. Therefore, the sooner the disease is diagnosed, the better. A safer and more effective diagnostic method is desirable. Biomarkers represent a potentially alternative method for diagnosis.

Proteomic technologies have enabled the identification of novel protein biomarkers [3]. Isobaric tags for relative and absolute quantification (iTRAQ) is a technique developed by the Applied Biosystems Incorporation [4]. In the present study, iTRAQ was used to analyse the total protein content of serum from patients with LT. iTRAQ was combined with Gene Ontology (GO) and pathway analysis to identify proteins that are differentially expressed in LT and determine their predicted functions.

## RESULTS

### Protein identification

In this iTRAQ quantification project, there were 5 homogeneous samples named A, B, C, D, E, and we performed 1 technical duplicate experiment. In total, 401198 spectrums were generated, and 6173 peptides and 1399 proteins were identified with the following cutoff: Mascot Percolator [5] Q-value ≤ 0.01 (more details on the method page of the MS/MS Ion Search).

### Protein quantification

These proteins, from the B-VS-A, were divided into 112 upregulated by ratio_B_114/A_113>1.2 (Table 1). Twenty-two proteins were identified, which were divided by a ratio >2.

**Table 1:** The upregulated proteins in serum samples of liver transplantation (Part of the data)

No.	Protein_ID	Description	Ratio_B_114/A_113
**1**	tr|J3QRK0|J3QRK0_HUMAN	Integrin beta-4 (Fragment) OS=Homo sapiens GN=ITGB4 PE=1 SV=1	10
**2**	tr|B4DUI8|B4DUI8_HUMAN	cDNA FLJ52761, highly similar to Actin, aortic smooth muscle OS=Homo sapiens PE=2 SV=1	10
**3**	sp|Q3SY84|K2C71_HUMAN	Keratin, type II cytoskeletal 71 OS=Homo sapiens GN=KRT71 PE=1 SV=3	10
**4**	sp|Q8IYL3|CA174_HUMAN	UPF0688 protein C1orf174 OS=Homo sapiens GN=C1orf174 PE=1 SV=2	10
**5**	tr|J3KSQ2|J3KSQ2_HUMAN	Clathrin heavy chain 1 (Fragment) OS=Homo sapiens GN=CLTC PE=1 SV=1	9.594
**6**	tr|A8K6A5|A8K6A5_HUMAN	cDNA FLJ77742, highly similar to Homo sapiens integrin, alpha 5 (fibronectin receptor, alpha polypeptide), mRNA OS=Homo sapiens PE=2 SV=1	9.575
**7**	tr|Q86UW0|Q86UW0_HUMAN	Ovarian epithelial carcinoma-related protein OS=Homo sapiens PE=2 SV=1	8.757
**8**	tr|A0A024R2T8|A0A024R2T8_HUMAN	Endonuclease G-like 1, isoform CRA_b OS=Homo sapiens GN=ENDOGL1 PE=4 SV=1	7.551
**9**	tr|D6RGG3|D6RGG3_HUMAN	Collagen alpha-1(XII) chain OS=Homo sapiens GN=COL12A1 PE=1 SV=1	6.66
**10**	sp|P25815|S100P_HUMAN	Protein S100-P OS=Homo sapiens GN=S100P PE=1 SV=2	5.38
**11**	sp|Q7L523|RRAGA_HUMAN	Ras-related GTP-binding protein A OS=Homo sapiens GN=RRAGA PE=1 SV=1	4.567
**12**	tr|Q7Z3Y6|Q7Z3Y6_HUMAN	Rearranged VH4-34 V gene segment (Fragment) OS=Homo sapiens GN=VH4-34 PE=4 SV=1	4.433
**13**	tr|A0A0C4DH43|A0A0C4DH43_HUMAN	Uncharacterized protein (Fragment) OS=Homo sapiens PE=4 SV=1	2.93
**14**	tr|Q9UL79|Q9UL79_HUMAN	Myosin-reactive immunoglobulin light chain variable region (Fragment) OS=Homo sapiens PE=2 SV=1	2.865
**15**	sp|Q9UM07|PADI4_HUMAN	Protein-arginine deiminase type-4 OS=Homo sapiens GN=PADI4 PE=1 SV=2	2.683
**16**	tr|B4E380|B4E380_HUMAN	Histone H3 OS=Homo sapiens PE=2 SV=1	2.605
**17**	tr|D6RF35|D6RF35_HUMAN	Vitamin D-binding protein OS=Homo sapiens GN=GC PE=1 SV=1	2.355
**18**	tr|B1AH77|B1AH77_HUMAN	Ras-related C3 botulinum toxin substrate 2 OS=Homo sapiens GN=RAC2 PE=1 SV=1	2.195
**19**	sp|P62805|H4_HUMAN	Histone H4 OS=Homo sapiens GN=HIST1H4L PE=2 SV=1	2.172
**20**	tr|Q5NV63|Q5NV63_HUMAN	V1-4 protein (Fragment) OS=Homo sapiens GN=V1-4 PE=4 SV=1	2.092
**21**	sp|P68871|HBB_HUMAN	Hemoglobin, beta OS=Homo sapiens GN=HBB PE=3 SV=1	2.081
**22**	tr|D6R9C5|D6R9C5_HUMAN	Osteopontin (Fragment) OS=Homo sapiens GN=SPP1 PE=1 SV=1	2.078

### COG annotation for all identified proteins

**Figure 1:**
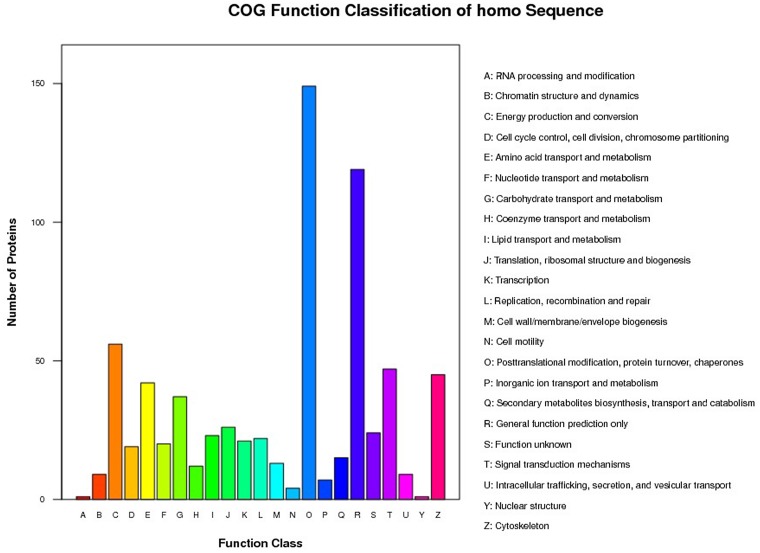
Histogram of the GOG Analysis x-axis displays the COG term, y-axis displays the corresponding protein count illustrating the protein number of different function.

All of the identified proteins were classified into different COG functional categories and subsequently into 23 subcategories (Figure 1). Most of these proteins were involved in “post-translational modification, protein turnover, chaperones” and in “general function prediction only”.

### Pathway enrichment analysis of DEPs

**Figure 2:**
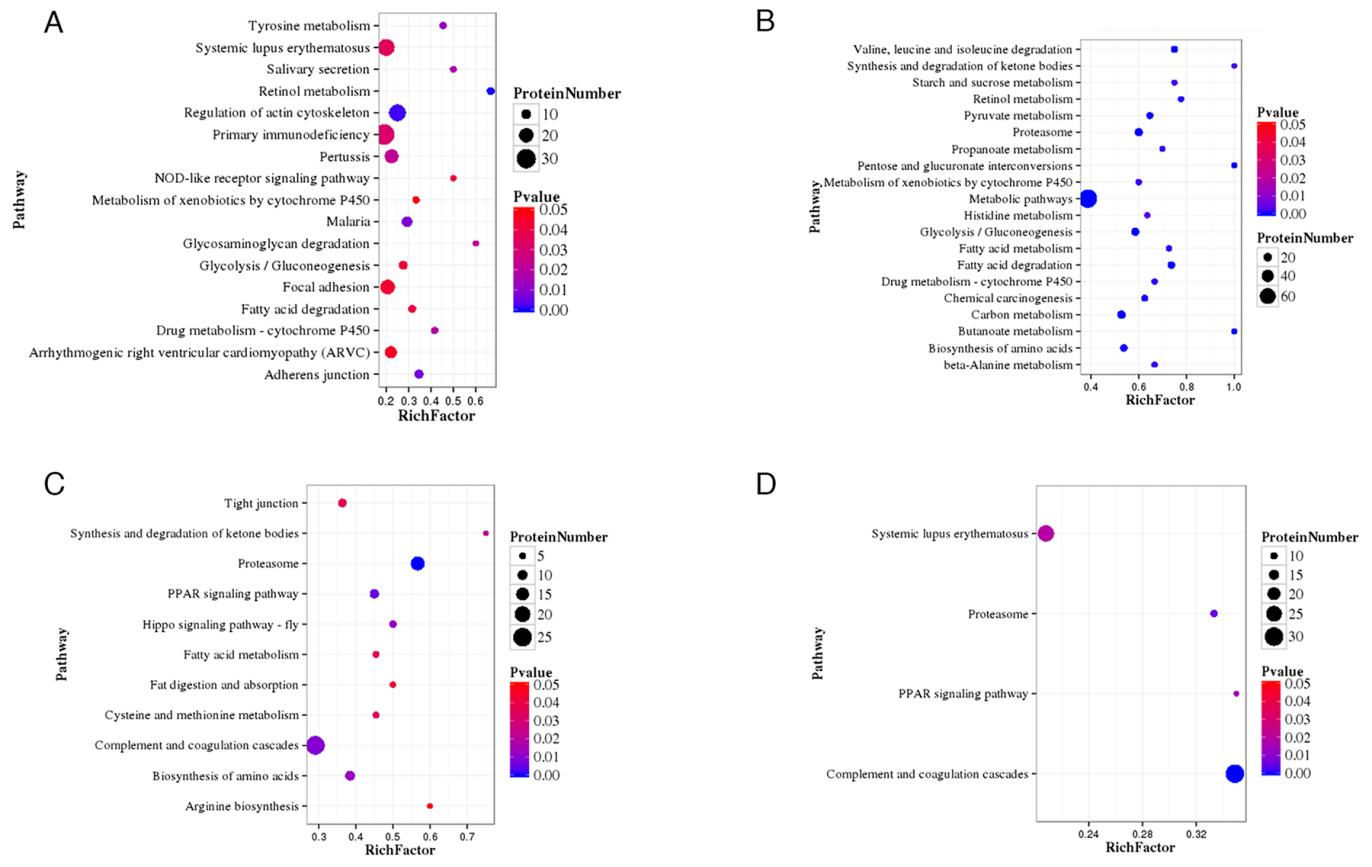
**(A)**:class B vs class A; **(B):**class C vs class A; **(C):**class D vs class A; **(D):**class E vs class A Statistics of pathway enrichment of differentially expressed proteins in each pairwise. RichFactor is the ratio of differentially expressed protein number annotated in this pathway term to all protein number annotated in this pathway term. Greater richFator means greater intensiveness. Pvalue ranges from 0~1, and less Pvalue means greater intensiveness.

**Figure 3:**
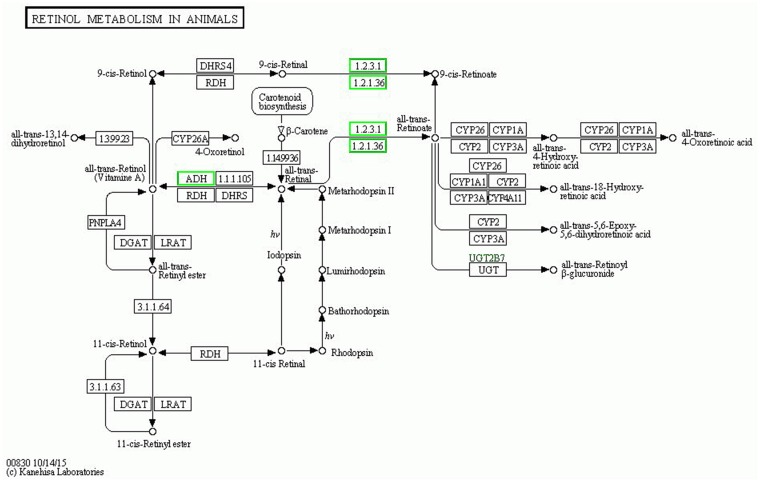
The KEGG pathway of “retinol metabolism” Down-regulated proteins are marked with with green borders and non-change proteins are marked with black borders.

Data from Figure 1 can be compared with the data in Figure 2, which shows that the P-value of retinol metabolism is the lowest in the B-VS-A pathway. The rich factor is the ratio of differentially expressed protein number annotated in this pathway to all proteins annotated in this pathway. A greater rich factor value corresponds to a greater intensiveness. The P-value ranges from 0~1, and a lower P-value indicates greater intensiveness (Figure 2). The KEGG pathway of retinol metabolism is shown in Figure 3. Perisinusoidal stellate cells of the liver play important roles in retinol metabolism and fibrosis [6].

There are 6 differentially expressed proteins annotated in retinol metabolism (Table 2), which are alcohol dehydrogenase 1A; epididymis luminal protein 9; alcohol dehydrogenase IB (Class I), beta polypeptide, isoform CRA_a; alcohol dehydrogenase 6; aldehyde oxidase and alcohol dehydrogenase 4.

**Table 2:** differentially expressed protein annotated in retinol metabolism

No.	Protein_ID	Description	Ratio_B_114/A_113
**1**	sp|P00325|ADH1B_HUMAN	Alcohol dehydrogenase IB (Class I), beta polypeptide, isoform CRA_a OS=Homo sapiens GN=ADH1B PE=4 SV=1	0.299
**2**	sp|P07327|ADH1A_HUMAN	Alcohol dehydrogenase 1A OS=Homo sapiens GN=ADH1A PE=1 SV=2	0.399
**3**	sp|P08319|ADH4_HUMAN	Alcohol dehydrogenase 4 OS=Homo sapiens GN=ADH4 PE=1 SV=5	0.438
**4**	sp|Q06278|AOXA_HUMAN	Aldehyde oxidase OS=Homo sapiens GN=AOX1 PE=1 SV=2	0.68
**5**	sp|P28332|ADH6_HUMAN	Alcohol dehydrogenase 6 OS=Homo sapiens GN=ADH6 PE=1 SV=2	0.721
**6**	tr|V9HVX6|V9HVX6_HUMAN	Epididymis luminal protein 9 OS=Homo sapiens GN=HEL-9 PE=2 SV=1	0.808

## DISCUSSION

Quantification of the proteins via iTRAQ analysis has been suggested to be a suitable strategy for the identification of biomarkers since iTRAQ allows the comparison of protein abundance among samples by measuring the peak intensities of reporter ions released from the iTRAQ-tagged peptides. In this study, iTRAQ was combined with Gene Ontology (GO) and pathway analysis to identify the proteins that are differentially expressed in LT and determine their predicted functions.

Among the differentially expressed proteins in Table 1 and based on published studies, protein S100-P was found to be relevant to liver cancer. However, in the other groups, C-VS-A_up, D-VS-A_up and E-VS-A_up, S100-P was not significantly upregulated.

S100 calcium-binding protein P (S100P) is a protein that is encoded by the S100P gene in humans [7]. The protein encoded by this gene is a member of the S100 family of proteins containing 2 EF-hand calcium-binding motifs. S100 proteins are localized in the cytoplasm and/or nucleus of a wide range of cells and are involved in the regulation of a number of cellular processes, such as cell cycle progression and differentiation. S100 genes include at least 13 members that are located as a cluster on chromosome 1q21; however, this gene is located at 4p16. This protein, in addition to binding Ca2+, also binds Zn2+ and Mg2+. This protein may play a role in the aetiology of prostate cancer [8]. S100P regulates the calcium signal transduction pathway to mediate cytoskeletal interactions and is induced by the prostaglandin E2 (PGE2)/EP4) receptor signalling pathway in colon cancer cells [9]. The ectopic expression of S100P in SW 480 CRC promotes invasion and metastasis and decreases sensitivity to 5-FU *in vitro* [10]. The results of Hui Dong et al. indicated that S100P was upregulated in HCC, suggesting a possible role for S100P in liver cancer. The increased expression of both CCL20 and S100P genes was detected in HCC by quantitative RT-PCR, although they were found to be significantly upregulated in HepG2 but not in HCC SAGE data [11].

In this study, we found that the concentration of S100P in the serum before transplantation (class B) was 5.38x higher than that of the control group (class A). The GO biological processes associated with S100P were cellular process, localization, locomotion, response to stimulus and single-organism process. The GO cellular components associated with S100P were cell part and organelle. The GO molecular function associated with S100P was binding.

Aldehyde oxidase (AO) is a metabolizing enzyme and plays a very important role in the metabolization of numerous drugs. AO catalyses the oxidation of aldehydes into carboxylic acid and catalyses the hydrolysation of some heterocycles. In addition, AO can catalyse the oxidation of both cytochrome P450 (CYP450) and monoamine oxidase (MAO) intermediate products [12]. However, in the other groups, C-VS-A_up, D-VS-A_up and E-VS-A_up, S100-P was not significantly upregulated.

We found that the concentration of AO in the serum of patients before transplantation (class B) was 1.47x lower than that of the control group (class A). The GO biological process associated with AO were cellular process, metabolic process, and response to stimulus. The GO cellular component associated with AO was cell parts. The GO molecular function associated with AO were binding, catalytic activity, and electron carrier activity.

In conclusion, iTRAQ technology, which represents a relatively new strategy for proteomic analysis, was used here to study the changes in protein expression associated with liver cancer. This approach identified 2 significantly differentially expressed proteins (S100P and aldehyde oxidase) that were previously reported to be related to liver cancer. The MFs, CCs and BPs associated with these proteins indicate that they may be suitable liver biomarkers, which may improve the timeliness and accuracy of diagnosis. However, further investigation of the functions of these proteins, as well as of the proteins with high fold changes in liver cancer identified herein, is needed.

## MATERIALS AND METHODS

Samples. Serum samples were obtained from liver transplant patients between October 2014 and August 2015 at the Guilin 181^st^ Hospital (Guilin No. 181 Hospital officially changed its name to Guilin No. 924 Hospital), Guangxi, China. All patients were transplanted for HCC.

Serum samples were collected from 9 healthy volunteers who served as controls (class A) and from 9 liver transplant patients at different timepoints as follows: before transplantation (class B) and after transplantation on the 1st (class C), the 3rd (class D) and the 7th (class E) days. The main clinical and biochemical characteristics of liver transplant patients and the control group are shown in Table 3.

**Table 3:** Main clinical and biochemical characteristics of patients with liver transplant and the control group

	class A	class B	class C	class D	class E
Peripheral samples (n)	9	9	9	9	9
Ages (years)	44±12	47±15	47±15	47±15	47±15
Sex(male/female)	8/1	8/1	8/1	8/1	8/1
ALT (IU/L)	25.3±9.0	80.2±20.1	315.7±60.3	178.5±39.8	81.1±29.4
AST(IU/L)	21.7±7.9	65.5±22.3	293.8±62.5	161.4±41.5	69.5±31.8

This study was performed according to the guidelines set forth by the Guilin 181st Hospital and abides by the Declaration of Helsinki on ethical principles for medical research involving human subjects. Written informed consent was obtained from all the subjects or their guardians.

### Sample preparation

Serum (5 ml) was collected from the enrolled subjects in a coagulation tube; the serum was separated, and 100 μl aliquots were stored at -80°C until further use.

### iTRAQ

The concentration of the extracted protein was measured using the Bradford method. According to the concentration results, 30 μg of protein was removed from each sample for electrophoresis. Then, 100 μg of protein was accurately removed for digestion from each sample solution.

After trypsin digestion, the peptides were vacuum centrifuged to dryness. The iTRAQ labelling of peptide samples was performed using the iTRAQ Reagent 8-plex Kit according to the manufacturer's protocol. The peptides labelled with respective isobaric tags were incubated for 2 h. The iTRAQ-labelled peptides were fractionated using RP.

### Peptide fractionation by RP chromatography

For RP chromatography using the Shimadzu LC-20AB HPLC Pump System, the peptides from digestion were reconstituted with buffer A (5% acetonitrile, 95% H2O, adjusted to pH 9.8 with ammonia) to 2 ml and loaded onto a 4.6×250 mm Gemini C18 column containing 5 μm particles (Phenomenex). The peptides were eluted at a flow rate of 1 mL/min with a gradient of 5% buffer B (5% H2O, 95% acetonitrile, adjusted to pH 9.8 with ammonia) for 10 min, 5-35% buffer B for 40 min, and 35-95% buffer B for 1 min. The system was then maintained in 95% buffer B for 3 min and decreased to 5% within 1 min before equilibrating with 5% buffer B for 10 min. Elution was monitored by measuring absorbance at 214 nm, and fractions were collected every 1 min. The eluted peptides were pooled as 20 fractions and vacuum-dried.

### LC-ESI-MS/MS analysis based on Triple TOF 5600

Each fraction was resuspended in buffer A (5% acetonitrile, 0.1% FA) and centrifuged at 20,000 × g for 10 min, and the final concentration of peptide was approximately 0.5 mg/mL. The supernatant was loaded on a LC-20AD Nano HPLC (Shimadzu, Kyoto, Japan) by the autosampler onto a 2 cm C18 trap column. Then, the peptides were eluted into a 18 cm analytical C18 column (inner diameter 75 mm, packed in-house). The samples were loaded at 8 mL/min for 4 min, continued by a 41 min gradient running at 300 nL/min from 5 to 35% buffer B (95% acetonitrile, 0.1% FA), followed by a 5 min linear gradient to 80% buffer B; the samples were maintained at 80% for 5 min and finally returned to 5% for 1 min.

### Bioinformatic pipeline

After the quantification of proteins, all the proteins with a false discovery rate (FDR) of less than 1% proceeded with the downstream analysis, including GO, COG and Pathway. Further, we performed deep analysis based on differentially expressed proteins, including Gene Ontology (GO) enrichment analysis, KEGG pathway enrichment analysis and cluster analysis.

## References

[1] Affo S, Yu L, Schwabe RF (2017). The role of cancer-associated fibroblasts and fibrosis in liver cancer. Annu Rev Pathol.

[2] Pinheiro RS, Waisberg DR, Nacif LS, Rocha-Santos V, Arantes RM, Ducatti L, Martino RB, Lai Q, Andraus W, D'Albuquerque LAC (2017). Living donor liver transplantation for hepatocellular cancer: An (almost) exclusive Eastern procedure?. Transl Gastroenterol Hepatol.

[3] Sui W, Zhang R, Chen J, He H, Cui Z, Ou M, Li W, Qi S, Wen J, Lin X, Dai Y (2015). Quantitative proteomic analysis of Down syndrome in the umbilical cord blood using iTRAQ. Mol Med Rep.

[4] Sun C, Song C, Ma Z, Xu K, Zhang Y, Jin H, Tong S, Ding W, Xia G, Ding Q (2011). Periostin identified as a potential biomarker of prostate cancer by iTRAQ-proteomics analysis of prostate biopsy. Proteome Sci.

[5] Brosch M, Yu L, Hubbard T, Choudhary J (2009). Accurate and sensitive peptide identification with mascot percolator. Journal of Proteome Research.

[6] Blomhoff R, Wake K (1991). Perisinusoidal stellate cells of the liver: important roles in retinol metabolism and fibrosis. FASEB J.

[7] Schäfer BW, Wicki R, Engelkamp D, Mattei MG, Heizmann CW (1995). Isolation of a YAC clone covering a cluster of nine S100 genes on human chromosome 1q21: rationale for a new nomenclature of the S100 calcium-binding protein family. Genomics.

[8] https://www.ncbi.nlm.nih.gov/gene?Db=gene&Cmd=Show DetailView&TermToSearch=6286

[9] Chandramouli A, Mercado-Pimentel ME, Hutchinson A, Gibadulinová A, Olson ER, Dickinson S, Shañas R, Davenport J, Owens J, Bhattacharyya AK, Regan JW, Pastorekova S, Arumugam T (2010). The induction of S100p expression by the Prostaglandin E2 (PGE2)/EP4 receptor signaling pathway in colon cancer cells. Cancer Biol Ther.

[10] Dong L, Wang F, Yin X, Chen L, Li G, Lin F, Ni W, Wu J, Jin R, Jiang L (2014). Overexpression of S100P promotes colorectal cancer metastasis and decreases chemosensitivity to 5-FU *in vitro*. Mol Cell Biochem.

[11] Dong H, Ge X, Shen Y, Chen L, Kong Y, Zhang H, Man X, Tang L, Yuan H, Wang H, Zhao G, Jin W (2009). Gene expression profile analysis of human hepatocellular carcinoma using SAGE and LongSAGE. BMC Med Genomics.

[12] https://en.wikipedia.org/wiki/Aldehyde_oxidase

